# Effects of Various Doses of Caffeine Ingestion on Intermittent Exercise Performance and Cognition

**DOI:** 10.3390/brainsci10090595

**Published:** 2020-08-28

**Authors:** Cuicui Wang, Yuechuan Zhu, Cheng Dong, Zigui Zhou, Xinyan Zheng

**Affiliations:** School of Kinesiology, Shanghai University of Sport, Shanghai 200438, China; 1821516026@sus.edu.cn (C.W.); 1821516044@sus.edu.cn (Y.Z.); 1821518006@sus.edu.cn (C.D.); 1921516023@sus.edu.cn (Z.Z.)

**Keywords:** caffeine, prolonged intermittent exercise, cognition, exercise performance

## Abstract

To date, no study has examined the effects of caffeine on prolonged intermittent exercise performance that imitates certain team-sports, and the suitable concentration of caffeine for improved intermittent exercise performance remains elusive. The purpose of the present cross-over, double-blind preliminary study was to investigate effects of low, moderate, and high doses of caffeine ingestion on intermittent exercise performance and cognition. Ten males performed a familiarization session and four experimental trials. Participants ingested capsules of placebo or caffeine (3, 6, or 9 mg/kg) at 1 h before exercise, rested quietly, and then performed cycling for 2 × 30 min. The cycling protocol consisted of maximal power pedaling for 5 s (mass × 0.075 kp) every minute, separated by unloaded pedaling for 25 s and rest for 30 s. At pre-ingestion of capsules, 1 h post-ingestion, and post-exercise, participants completed the Stroop task. The mean power-output (MPO), peak power-output (PPO), and response time (RT) in the Stroop task were measured. Only 3 mg/kg of caffeine had positive effects on the mean PPO and MPO; 3 mg/kg caffeine decreased RTs significantly in the incongruent and congruent conditions. These results indicate that the ingestion of low-dose caffeine had greater positive effects on the participants’ physical strength during prolonged intermittent exercise and cognition than moderate- or high-dose caffeine.

## 1. Introduction

As a drug from the methylxanthine family, caffeine ingestion is highly prevalent not only in the general population but also among athletes [1]. In 2004, caffeine was removed from the list of banned substances by the World Anti-Doping Agency and was reaffirmed as a regulatory drug [2]. Since then, many athletes ingest caffeine to improve their exercise performance. Caffeine has shown an ergogenic effect on endurance-based exercise [3,4,5,6,7,8]. In a study investigating the effects of three different doses of caffeine on prolonged exercise capacity, Graham and Spriet [9] found that the ingestion of a low (3 mg/kg) or a moderate (6 mg/kg) dose of caffeine delayed the time to exhaustion, whereas a high dose (9 mg/kg) did not. These studies indicate that the beneficial effects of caffeine on endurance exercise can be achieved at a low-to-moderate dose, with 6 mg/kg caffeine often suggested. Moreover, lower doses of caffeine do not affect peripheral whole-body responses to exercise and are associated with few, if any, side effects. Spriet [10] suggested low doses of caffeine ingestion for improving exercise performance. Therefore, it is necessary to clarify whether other types of exercise can benefit from a suitable concentration of caffeine.

A successful performance of intermittent exercise is greatly related to an athlete’s ability to perform repeated bouts of high-intensity sprint exercises [11]. Therefore, many researchers have studied the effects of caffeine on repetitive high-intensity exercise. For example, Beaven et al. [12] studied 12 trained males who performed 5 × 6 s sprints interspersed with 24 s of active recovery on a cycle ergometer after the ingestion of caffeine. They found that a 1.2% caffeine solution significantly improved the maximal exercise performance. Another study revealed an increase in the total amount of sprint work and the mean peak power output (PPO) following caffeine supplementation (6 mg/kg), compared with a placebo, during an intermittent sprint test consisting of 2 × 36 min halves, each composed of 18 × 4 s sprints with a 2 min active recovery at 35% of the peak volume of oxygen between each sprint [13]. Using a similar exercise protocol, Crowe et al. [14] demonstrated that the ingestion of 6 mg/kg of caffeine did not improve the results of repeated 60-s maximal cycling tests with a 30 s rest between each exercise. In addition, Salinero et al. [15] reported that caffeine ingestion (3 mg/kg) increased both the PPO and the mean power output (MPO) during the Wingate test in a group of young men and women. Above all, the effects of caffeine on prolonged intermittent exercise performance are inconsistent, and the suitable caffeine concentration for improved intermittent exercise performance remains elusive.

The improvement of exercise performance not only represents the enhancement of physical strength but also includes the development of psychological and cognitive functions necessary in sports [16]. Specially in some team sports, most points are scored in the latter stages of the match; however, the development of fatigue, particularly in the latter half, contributes to decreasing concentration, executive information processing, and decision making [17]. The ergogenic effect of caffeine might not only enhance physical strength but also the development of cognitive functions during exercise. As a stimulant drug, caffeine may have positive effects on cognition [18,19]. Cognition encompasses a great variety of mental processes, including those mediating executive functioning, decision-making, and creativity. Executive functioning is important for athletic performance and can be affected by prolonged physical exertion [20]. Research regarding the effects of caffeine consumption on performance in the Stroop task, a measure of executive function, has been inconsistent. Hogervorst et al. [18] reported that the ingestion of 150 mg of caffeine effectively accelerated response time (RT) in the Stroop task during and after exercise. In addition, Ali et al. [21] observed that a caffeine dose of 6 mg/kg effectively decreased RT in the Stroop task among female football players. However, another study found that caffeine did not have an effect on the Stroop performance after exercise [22]. The differences in these results may be related to the sensitivity of participants to various exercise types, cognitive tests, or caffeine doses. Therefore, it is necessary to compare the effects of different concentrations of caffeine on cognitive function and clarify the optimal concentration of caffeine required to improve cognitive function.

The purpose of the present preliminary study was to investigate the effects of low (3 mg/kg), moderate (6 mg/kg), and high (9 mg/kg) doses of caffeine ingestion on intermittent exercise performance and cognitive performance. We hypothesized that the ingestion of a low dose of caffeine would enhance intermittent exercise performance with a corresponding decrease in RT and the rating of perceived exertion (RPE) compared with the placebo and other doses of caffeine.

## 2. Materials and Methods

### 2.1. Participants

Ten healthy, low-caffeine-consuming male soccer players (age: 20.88 ± 2.72 years, height: 176.7 ± 5.1 cm, weight: 72.1 ± 8.7 kg) participated in this study. The sample size used was based on a G*Power 3.1 software calculation (effect size = 0.15) [23,24]. The participants were deemed eligible for this study if their caffeine intake was less than 60 mg/day (did not consume coffee, tea, caffeine-containing energy drinks or supplements, or chocolate; and consumption of cola <330 mL a day), they exercised at least three times/week, they had no injury or surgery in the past six months, they had a normal cognitive function (Mini Mental State Examination score ≥ 26), they were right-handed, and they could perform high-intensity exercise and had normal executive functions. The participants were fully informed of any risks and discomforts associated with the experiment before they provided their informed written consent to participate. The study followed the guidelines of the Declaration of Helsinki and was approved by the local ethics committee at Shanghai University in Sport, Shanghai, China (No. 2016008).

### 2.2. Procedures

The participants visited the laboratory five times, including one familiarization trial and four experimental trials. All participants completed all experimental conditions at the same time of the day and at least 2 h after eating to minimize circadian-type variance in body temperature and other biological variables. Meanwhile, in order to better simulate the practical game, the exercise time was in line with the game time at about 3:00 in the afternoon; the exposure to each condition was separated by 1 week to ensure drug washout. The participants abstained from alcohol, food, or drinks containing caffeine (i.e., coffee, tea, cola, energy drinks, caffeine-containing supplements, chocolate), and strenuous exercise for 24 h before the experiment. During the first visit, they were familiarized with the equipment and procedures involved in the study. The participants adjusted the seat and bar heights and positions of their cycle simulator and replicated these positions in the four subsequent experimental exercise trials. After the exercise, the food consumed by each participant during 24 h before the familiarizing experiment was recorded. Participants were asked to replicate this diet prior to subsequent trials.

On the day of the experiment, the participants were asked to go to the toilet and empty their bladder, and then they had their body weight and height measured. The participants were seated in a comfortable chair for the cognitive tasks (Stroop tasks). The Stroop tasks consisted of one practice trial and one baseline (Stroop pre) trial. Following that, the participants ingested capsules containing placebo (calcium carbonate; CON), 3 mg/kg (CAF3), 6 mg/kg (CAF6), or 9 mg/kg (CAF9) of caffeine with 200 mL of water. After a 40-min seated rest, the participants performed the Stroop task, walked to a cycle simulator, and prepared to exercise.

The participants warmed up by cycling for 5 min (body mass × 0.01 kp) and then rested for 5 min. It has been reported that the plasma caffeine concentration is maximal 60 min after ingestion of caffeine [7]. At 1 h after the drug administration, they began to complete a laboratory-based intermittent exercise protocol designed to replicate the demands of an actual sports game [25]. The protocol consisted of two 30-min halves separated by a 15 min half-time break, with each half consisting of two trials separated by a 2 min rest period. One set consisted of maximal pedaling (body mass × 0.075 kp) for 5 s, active recovery (no load, 80 rpm) for 25 s, and resting for 30 s. During the 5 s maximal pedaling, to maintain their effort, verbal encouragement was provided throughout each bout. One trial consisted of 15 sets. The participants performed a total of four trials via a cycling ergometer (Monark 839E, Monark Exercise AB, Vansbro, Sweden). The PPO and MPO were recorded for each 5-s loaded sprint. Finally, the Stroop tasks were repeated. All participants completed all experimental conditions in the normal environment (23 °C, 50% relative humidity, Second Multi air conditioning system, Fuji Medical Science Co. LTD, Tokyo, Japan), and exposures were separated by 1 week to ensure drug washout.

The RPE was recorded at 3 min intervals throughout the cycling process. The Borg 6–20 RPE scale was printed onto a piece of paper, placed on a clipboard, and held in front of each participant when needed during each trial [26]. The heart rate (HR) was monitored via a HR monitor (model RS400; Polar Electro Oy, Kemple, Finland) during the entire process of the experimental trial.

### 2.3. Drug Treatment

A cross-over, double-blind design was used in the present study. Caffeine hydrate and calcium carbonate were obtained as white powders (034-06782, Wako Pure Chemical Industries, Ltd., Osaka, Japan). The dosages were calculated according to each participant’s body weight. The treatments, each of which was delivered in three red capsules, were as follows: CON, 9 mg/kg calcium carbonate; CAF3, a mixture of 3 mg/kg caffeine and 6 mg/kg calcium carbonate; CAF6, a mixture of 6 mg/kg caffeine and 3 mg/kg calcium carbonate; and CAF9, 9 mg/kg caffeine. The researchers and participants could not identify the caffeine dosage by the appearance or taste of the capsules.

### 2.4. Stroop Task

The Stroop task is widely used to evaluate selective attention, cognitive flexibility, and processing speed [27]. The task was programmed and performed using E-prime 1.0 software (Psychology Software Tools, Pittsburgh, PA, USA). Each trial was displayed as follows: a fixed cross in the center of the screen for 500 ms, followed by a 500-ms stimulus. There were two kinds of stimuli: congruent and incongruent. In the congruent condition, three Chinese color words were shown (绿 for green, 蓝 for blue, and 红 for red), with the font colors matching the colors of each word. In the incongruent condition, the same three words were presented, but the font color did not match the color indicated by the word (e.g., the word “green” was presented in blue or red font). The participants were required to indicate the presentation color of each word on a numeric keypad, wherein the 1, 2, and 3 keys corresponded to the responses of blue, green, and red, respectively. The participants used their index, middle, and ring fingers of their right hand to press the keys, which were situated in the left-to-right order of 1, 2, and 3. The RT and accuracy rate (ACC) were measured.

The participants performed two blocks of 120 trials at pre-ingestion of the capsules, 60 min post-ingestion of the capsules, and post-exercise. Each block included 60 congruent and 60 incongruent trials, which were randomly presented. To prevent the participants from anticipating the stimulus, the interval between the appearance of the fixed cross and the presentation of the stimulus was randomly changed between 300 and 800 ms, with a fixed interstimulus interval duration of 1500 ms. The values for both the RT and ACC were recorded for further analysis.

### 2.5. Statistical Analyses

All statistical calculations were conducted with SPSS 20.0 software (SPSS Inc., Chicago, IL, USA). The one-sample Kolmogorov–Smirnov test was used to test whether the data were normally distributed. When the data were not normally distributed, statistical analysis was performed on the logarithmic transformation of the data. Alterations in the mean PPO, MPO, RPE, HR, RT, and ACC values were subjected to a two-factor (condition × time) analysis of variance with repeated measures. For cases in which the assumption of sphericity was violated, the Greenhouse–Geisser correction was used to reduce the likelihood of a type I error. If significant main or interaction effects were found, post-hoc analyses were carried out with the Bonferroni correction. For analysis of variance (ANOVA), partial eta^2^ (Pη^2^) was used as a measure of the effect size. The criteria to interpret the magnitude of the effect size were as follows: small, Pη^2^ = 0.01; medium, Pη^2^ = 0.06; and large, Pη^2^ = 0.14 [28]. Data were summarized as the mean ± standard deviation. Statistical significance was accepted at *P* < 0.05.

## 3. Results

### 3.1. Exercise Performance

**Figure 1:**
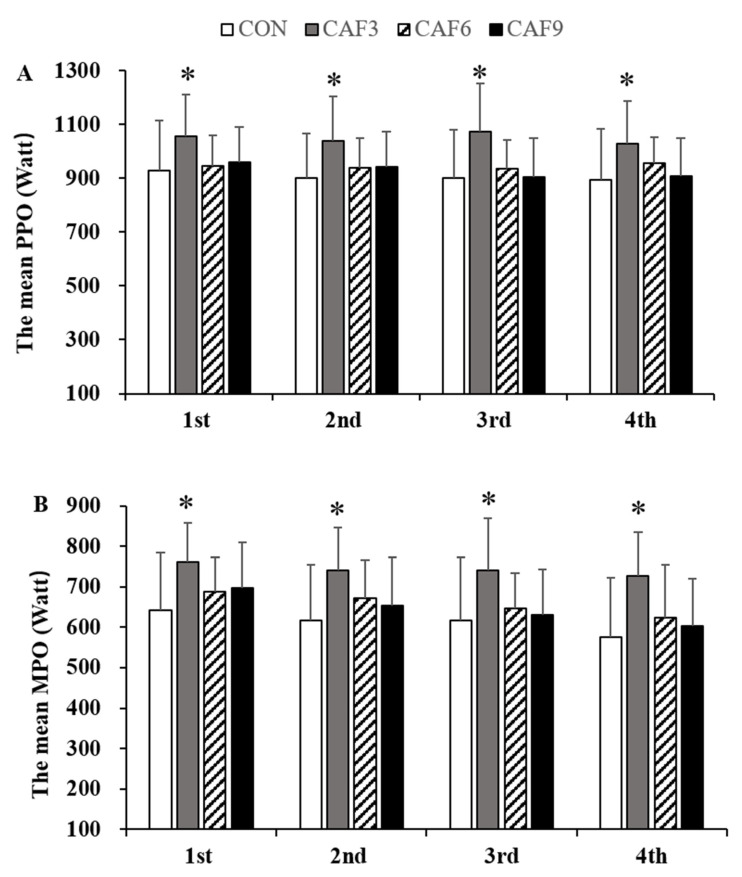
The peak power output (**A**) and mean power output (**B**) per trial. PPO, peak power output; MPO, mean power output; CON, placebo ingestion group; CAF3, 3 mg/kg caffeine ingestion group; CAF6, 6 mg/kg caffeine ingestion group; CAF9, 9 mg/kg caffeine ingestion group. *, vs. CON. Values are mean ± SD, *p* < 0.05.

For the mean PPO, a 4 × 4 mixed ANOVA revealed that there was no significant interaction (F (3, 9) = 1.12, *P* = 0.36, Pη^2^ = 0.111, Figure 1A), but there was a significant main effect of the condition (F (3) = 3.83, *P* < 0.05, Pη^2^ = 0.299). For all time points, the mean PPO in the CAF3 group was significantly greater than those in the other groups.

For the mean MPO, a 4 × 4 mixed ANOVA revealed that there was no significant interaction (F (3, 9) = 0.72, *p* = 0.58, Pη^2^ = 0.074, Figure 1B), but there was a significant main effect of the condition (F (3) = 7.73, *p* < 0.05, Pη^2^ = 0.518). For all conditions, the mean MPO in the CAF3 group was significantly greater than those in the other groups.

### 3.2. HR

**Figure 2:**
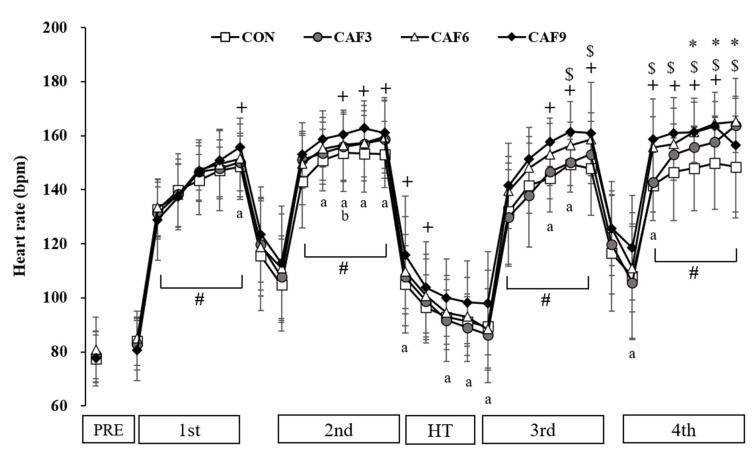
Change in heart rate. CON, placebo ingestion group; CAF3, 3 mg/kg caffeine ingestion group; CAF6, 6 mg/kg caffeine ingestion group; CAF9, 9 mg/kg caffeine ingestion group; PRE, pre ingestion drugs; HT, half time. +, CON vs. CAF9; $, CON vs. CAF6; *, CON vs. CAF3; a, CAF3 vs. CAF9; b, CAF6 vs. CAF9; #, significantly compared with PRE. Values are mean ± SD, *p* < 0.05.

For HR, a 4 × 33 mixed ANOVA revealed that there was no significant interaction (F (3, 96) = 2.038, *p* = 0.156, Pη^2^ = 0.337, Figure 2), but there were significant main effects of condition (F (3) = 5.57, *p* < 0.05, Pη^2^ = 0.582) and time (F (32) = 204.25, *p* < 0.001, Pη^2^ = 0.981). The HR changed over time during the exercise protocol. Moreover, all doses of caffeine ingestion induced a significant increase in the HR.

### 3.3. RPE

**Figure 3:**
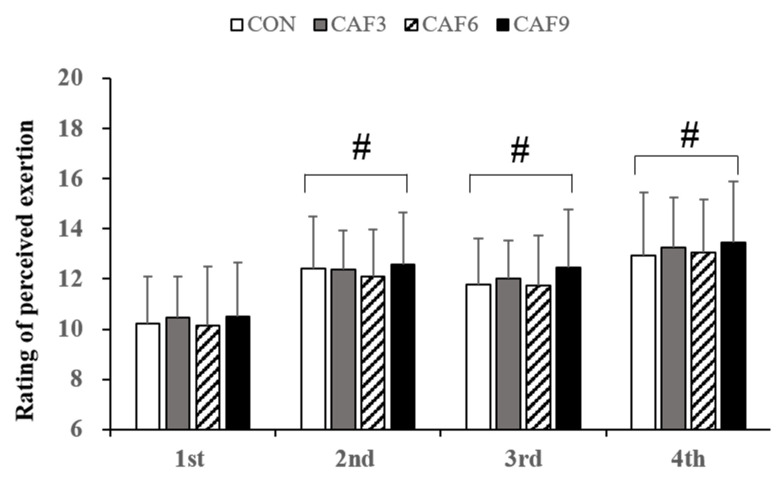
Change in rating of perceived exertion per trial. RPE, rating of perceived exertion; #, compared to 1st. Values are mean ± SD, *p* < 0.05.

Figure 3 summarizes the changes in the mean RPE per trial. For the mean RPE per trial, a 4 × 4 mixed ANOVA revealed that there was no significant interaction (F (3, 9) = 0.33, *p* = 0.83, Pη^2^ = 0.040), but there was a significant main effect of time (F (3) = 13.22, *p* < 0.001, Pη^2^ = 0.623). The RPE changed over time during the exercise protocol, but none of the caffeine doses affected the RPE.

### 3.4. Stroop Task: Incongruent Condition

For the RT in the Stroop task in the incongruent condition, a 4 × 3 mixed ANOVA revealed that there was a significant interaction (F (3, 6) = 3.5, *p* < 0.05, Pη^2^ = 0.28, Table 1). The RT at post-ingestion of the capsules in the CAF3 group was significantly faster than those in the other groups, and the RT at post-ingestion in the CAF6 group was significantly faster than those in the CON and CAF9 groups (CON: 603.78 ± 45.15 ms, CAF3: 564.68 ± 41.21 ms, CAF6: 582.75 ± 38.74 ms, CAF9: 609 ± 62 ms, *p* < 0.05). Furthermore, the RT at post-exercise in the CAF3 group was significantly faster than those in the CON and CAF9 groups (CON: 562.2 ± 30.79 ms, CAF3: 529.77 ± 35.94 ms, CAF6: 549.84 ± 37.82 ms, CAF9: 575.6 ± 38.37 ms, *p* < 0.05). For both the CAF3 and CAF6 groups, the RT was significantly faster at post-ingestion and post-exercise than at pre-ingestion. In all of the groups, there was a significantly faster RT at post-exercise compared with post-ingestion. For the ACC, the results from the Stroop task indicated no significant condition × time interaction (F (3, 6) = 0.61, *p* = 0.58, Pη^2^ = 0.337, Pη^2^ = 0.081, Table 1) or main effects of condition or time in the incongruent condition.

### 3.5. Stroop Task: Congruent Condition

For the RT in the Stroop task in the congruent condition, a 4 × 3 mixed ANOVA revealed that there was no significant interaction (F (3, 6) = 2.13, *p* = 0.11, Pη^2^ = 0.192, Table 1), but there was a significant main effect of time (F (2) = 25.88, *p* < 0.001, Pη^2^ = 0.742). For the CAF3 group, the RT was significantly faster at post-ingestion of the capsules and post-exercise compared with that at pre-ingestion (pre-ingestion: 582.61 ± 56.39 ms, post-ingestion: 537.15 ± 43.01 ms, post-exercise: 518.58 ± 36.69 ms, *p* < 0.05). Moreover, the RT was significantly faster at post-exercise compared with post-ingestion in the CON, CAF3, and CAF6 groups. For the ACC, the results from the Stroop task indicated no significant condition × time interaction (F (3, 9) = 0.93, *p* = 0.40, Pη^2^ = 0.117, Table 1) or main effects for condition or time in the congruent condition.

## 4. Discussion

To the best of our knowledge, no study has examined the effects of different doses of caffeine ingestion on prolonged intermittent exercise performance that imitates certain team sports, and the suitable ingestion concentration remains elusive. Data from the current preliminary study showed that only 3 mg/kg of caffeine had positive effects on the mean MPO and PPO, while 6 mg/kg or 9 mg/kg caffeine did not affect these values. These results indicate that the ingestion of a low dose of caffeine had greater positive effects on the participants’ physical strength during intermittent exercise than a moderate or high dose of caffeine.

To date, many studies have focused on the effects of caffeine ingestion on intermittent exercise performance [10,13,14,29,30,31,32,33,34,35]. In this study, we found that 3 mg/kg of caffeine had positive effects on the mean MPO and PPO. Consistent with our results, Paton et al. [34], Evans et al. [30], and Ranchordas et al. [35] also found that low-dose caffeine ingestion improved an athlete’s physical strength during intermittent exercise. Moreover, several groups have reported that 6 mg/kg of caffeine ingestion improves an athlete’s physical strength during intermittent exercise [10,13,31], but others have reported no effects of this dose of caffeine ingestion [14,29,32,33]. In this study, ingestion of 6 mg/kg of caffeine failed to affect the participants’ physical strength during intermittent exercise, which supports previous studies that this dose has no effects on intermittent exercise performance. The potential ergogenic effects of caffeine may be dependent on the recovery interval, and the ergogenic effects of caffeine may be greater with a long recovery interval [36,37]. If the recovery interval becomes longer than 6 s, a moderate dose of caffeine ingestion may improve exercise performance. Therefore, exercise protocols should be standardized in future studies investigating the effects of a drug on physical strength during intermittent exercise. Unfortunately, a high dose of caffeine failed to affect the participants’ physical strength during intermittent exercise and cognitive performance, which could be related, perhaps in part, to the adverse effects of caffeine, such as gastrointestinal upset, nervousness, mental confusion, and an impeded ability to focus [9]. Using the present experimental protocol, we showed that a low dose of caffeine had greater positive effects on the participants’ physical strength during intermittent exercise and cognitive performance than a moderate or high dose of caffeine. These results demonstrate that only low-dose caffeine ingestion can improve intermittent exercise performance imitating some team sports. Moreover, the peak plasma caffeine concentrations have been reported to reach maximal levels 60 to 90 min after ingestion [9], indicating that caffeine will exert some biological effects, but their timing might be different between individuals. In order to support our present conclusions, plasma caffeine concentrations should be measured in future studies.

Exercise performance improved by caffeine ingestion is presumed to be due to central nervous system stimulation, not peripheral mechanisms [38]. In the present study, we found that ingestion of 6 mg/kg or 9 mg/kg of caffeine increased the HR but failed to improve the intermittent exercise performance, while 3 mg/kg caffeine did not increase the HR but improved the intermittent exercise performance. These results support the conclusion by Davis and Green [39] that the ergogenic effects of caffeine depend on a mechanism involving the central nervous system, not multiple peripheral mechanisms. Caffeine acts as an antagonist of adenosine receptors, whereby the blockade of adenosine receptors, which have an inhibitory effect on neurons, causes neuronal excitation, enhances brain activation [40], attenuates the RPE [41], and improves cognition and physical ability during exercise. In the present study, we found that 3 mg/kg of caffeine ingestion enhanced the MPO and PPO but failed to affect the RPE. These results are consistent with those reported by Astorino et al. [42]. Their finding that the low dose of caffeine (2mg/kg) enhanced the intermittent exercise performance but did not change the RPE suggests that perceived exertion may be blunted by the low-dose caffeine intake [42]. Moreover, ingestion of 6 mg/kg or 9 mg/kg of caffeine did not affect the RPE or intermittent exercise performance in this study. A possible explanation of this result is that these doses failed to affect brain activation [41].

Previous studies focusing on aerobic exercise have shown that acute moderate aerobic exercise improves executive information processing related to selective attention and inhibitory control [21,43,44]. Different sports may have different effects on cognitive function; thus, recent studies have examined the effects of intermittent exercise on executive function. Consistent with the reports by Kujach et al. [45] and Ichinose et al. [46], the current study found that the RTs under conditions of incongruent or congruent stimuli were significantly shortened after intermittent exercise in all groups. These results indicate that the performance of prolonged intermittent exercise could also improve executive functioning.

By examining participants’ cognitive performance at pre-ingestion of the capsules, 60 min post-ingestion, and immediately post-exercise, this study was able to highlight the effects of different doses caffeine ingestion alone and post-exercise. Hogervorst et al. [47] found that participants were significantly faster after a low dose of caffeine ingestion (100 mg) on the computerized complex information processing test (Stroop Color–Word test) and Rapid Visual Information Processing Task, particularly after 140 min and after a time to the exhaustion trial. In the present study, we found that a low-dose caffeine ingestion improved incongruent and congruent conditions. These results are similar to Hogervorst et al. [47] and support that low-dose caffeine may have a direct and specific effect on perceptual-motor speed, efficiency factor, or executive information processing [48]. Moreover, the present finding of 6 mg/kg caffeine decreasing Stroop task RTs in the incongruent condition is consistent with Souissi et al. [49], whose findings showed that 6 mg/kg of caffeine improved RTs. These results suggest that the ingestion of a low or moderate dose of caffeine may reduce interference and thus improve exertive function during exercise. Our finding of no effect of high-dose caffeine on cognitive performance could be related, perhaps in part, to the adverse effects of caffeine. Given that the performance of our participants in both simple and complex task conditions was improved with the 3-mg/kg dose of caffeine, we suggest that low-dose caffeine may have an impact on cognition that is preferable to the effects of a moderate or high dose of caffeine. This hypothesis may be related to the increase in prefrontal cortex activation with lower doses of caffeine [40].

### Limitations

Although we used the G-power software to estimate the appropriate sample size, the number of participants only met the minimum sample size requirement. The present results should be replicated in future research with a larger sample size. Furthermore, the peak plasma caffeine concentration has been reported to reach maximal levels 15 to 120 min after ingestion, indicating that caffeine will exert some biological effects, but their timing might be different between individuals. However, in the present study, we did not measure the plasma levels of caffeine. Therefore, analyzing plasma levels of caffeine during intermittent exercise is necessary in future studies.

## 5. Conclusions

The results of this preliminary study indicate that the ingestion of a low dose of caffeine had greater positive effects on the participants’ physical strength during intermittent exercise and cognitive performance than a moderate or high dose of caffeine, suggesting that low-dose caffeine could improve intermittent exercise performance that imitates certain team sports.

## Figures and Tables

**Table 1:** Reaction time and accuracy rate of the Stroop task.

Measurements	Condition	Pre-Ingestion	Post-Ingestion	Post- Exercise
RT of incongruent (ms)	CON	604.85 ± 45.39	603.78 ± 45.15 *^,#^	562.20 ± 30.79 ^$^
	CAF3	614.98 ±50.56	564.68 ± 41.21 ^!^	529.77 ± 35.96 ^!,$^
	CAF6	630.38 ± 61.66	582.75 ± 38.74 *^,!^	549.84 ± 37.82 ^!,$^
	CAF9	623.96 ± 68.73	609.00 ± 62.00 *^,#^	575.60 ±38.37 ^$^
RT of congruent (ms)	CON	573.14 ± 32.76	573.08 ± 43.00	547.76 ± 38.09 ^$^
	CAF3	582.61 ± 56.39	537.15 ± 43.01 ^!^	518.58 ± 36.69 ^!,$^
	CAF6	582.31 ± 57.07	566.69 ± 43.99	531.93 ± 56.47 ^$^
	CAF9	577.45 ± 62.58	570.14 ± 45.18	544.71 ± 36.58 ^$^
ACC	CON	0.89 ± 0.08	0.91 ± 0.05	0.89 ± 0.07
of incongruent	CAF3	0.90 ± 0.07	0.90 ± 0.07	0.92 ± 0.06
	CAF6	0.87 ± 0.08	0.90 ± 0.05	0.90 ± 0.06
	CAF9	0.89 ± 0.08	0.91 ± 0.06	0.89 ± 0.11
ACC	CON	0.93 ± 0.04	0.91 ± 0.06	0.93 ± 0.04
of congruent	CAF3	0.93 ± 0.05	0.94 ± 0.04	0.95 ± 0.03
	CAF6	0.91 ± 0.11	0.94 ± 0.03	0.93 ± 0.06
	CAF9	0.92 ± 0.09	0.93 ± 0.04	0.92 ± 0.11

RT, reaction time; ACC, accuracy rate. !, significant vs. Pre-ingestion (*p* < 0.05); $, significant vs. Post-ingestion (*p* < 0.05); *, significant vs. CAF3 (*p* < 0.05); #, significant vs. CAF6 (*p* < 0.05). Values are mean ± SD.

## References

[1] Bishop D. (2010). Dietary supplements and team-sport performance. Sports. Med..

[2] Chester N., Wojek N. (2008). Caffeine consumption amongst british athletes following changes to the 2004 wada prohibited list. Int. J. Sports. Med..

[3] Pitchford N.W., Fell J.W., Leveritt M.D., Desbrow B., Shing C.M. (2014). Effect of caffeine on cycling time-trial performance in the heat. J. Sci. Med. Sport..

[4] Hodgson A.B., Randell R.K., Jeukendrup A.E., Earnest C.P. (2013). The metabolic and performance effects of caffeine compared to coffee during endurance exercise. PLoS ONE.

[5] Hoffmann R.W., Haeberlin E., Rohde T. (1995). The effect of different dosages of caffeine on endurance performance time. Int. J Sports Med..

[6] Desbrow B., Biddulph C., Devlin B., Grant G.D., Anoopkumar-Dukie S., Leveritt M.D. (2012). The effects of different doses of caffeine on endurance cycling time trial performance. J. Sports. Sci..

[7] Graham T.E. (2001). Caffeine and exercise: Metabolism, endurance and performance. Sports. Med..

[8] Cox G.R., Desbrow B., Montgomery P.G., Anderson M.E., Bruce C.R., Macrides T.A., Burke L.M. (2002). Effect of different protocols of caffeine intake on metabolism and endurance performance. J. Appl. Physiol..

[9] Graham T.E., Spriet L.L. (1995). Metabolic, catecholamine, and exercise performance responses to various doses of caffeine. J. Appl. Physiol..

[10] Spriet L.L. (2014). Exercise and sport performance with low doses of caffeine. Sports Med..

[11] Taylor J., Macpherson T., Spears I., Weston M. (2015). The effects of repeated-sprint training on field-based fitness measures: A meta-analysis of controlled and non-controlled trials. Sports. Med..

[12] Beaven C.M., Maulder P., Pooley A., Kilduff L., Cook C. (2013). Effects of caffeine and carbohydrate mouth rinses on repeated sprint performance. Appl. Physiol. Nutr. Metab..

[13] Schneiker K.T., Bishop D., Dawson B., Hackett L.P. (2006). Effects of caffeine on prolonged intermittent-sprint ability in team-sport athletes. Med. Sci. Sports Exerc..

[14] Crowe M.J., Leicht A.S., Spinks W.L. (2006). Physiological and cognitive responses to caffeine during repeated, high-intensity exercise. Int. J. Sport Nutr. Exerc. Metab..

[15] Salinero J., Lara B., Ruiz-Vicente D., Areces F., Puente-Torres C., Gallo-Salazar C., Coso J.D. (2017). CYP1A2 genotype variations do not modify the benefits and drawbacks of caffeine during exercise: A pilot study. Nutrients.

[16] Huang L., Deng Y., Zheng X., Liu Y. (2019). Transcranial direct current stimulation with Halo Sport enhances repeated sprint cycling and cognitive performance. Front. Physol..

[17] Bello M.L., Walker A.J., McFadden B.A., Sanders D.J., Arent S.M. (2019). Effects of TeaCrine and caffein on endurance and cognitive performance during a simulated match in high-level soccer players. J. Int. Soc. Sports. Nutr..

[18] Hogervorst E., Riedel W.J., Kovacs E., Brouns F., Jolles J. (1999). Caffeine improves cognitive performance after strenuous physical exercise. Int. J. Sports. Med..

[19] Smith A., Kendrick A., Maben A., Salmon J. (1994). Effects of breakfast and caffeine on cognitive performance, mood and cardiovascular functioning. Appetite.

[20] Yanagisawa H., Dan I., Tsuzuki D., Kato M., Okamoto M., Kyutoku Y., Soya H. (2010). Acute moderate exercise elicits increased dorsolateral prefrontal activation and improves cognitive performance with stroop test. Neuroimage.

[21] Ali A., O’Donnell J., Hurst P.V., Foskett A., Rutherfurd-Markwick K. (2016). Caffeine ingestion enhances perceptual responses during intermittent exercise in female team-game players. J. Sports..

[22] Bottoms L., Greenhalgh A., Gregory K. (2013). The effect of caffeine ingestion on skill maintenance and fatigue in epee fencers. J. Sports. Sci..

[23] Faul F., Erdfelder E., Lang A.G., Buchner A. (2007). G*Power 3: A flexible statistical power analysis program for the social, behavioral, and biomedical sciences. Behav. Res. Methods..

[24] Faul F., Erdfelder E., Buchner A., Lang A.G. (2009). Statistical power analyses using G*Power 3.1: Tests for correlation and regression analyses. Behav. Res. Methods.

[25] Chaen Y., Onitsuka S., Hasegawa H. (2019). Wearing a cooling vest during half-time improves intermittent exercise in the heat. Front. Physiol..

[26] Borg G.A. (1970). Perceived exertion as an indicator of somatic stress. Scand. J. Rehabil. Med..

[27] Pauw K.D., Roelands B., Knaepen K., Polfliet M., Stiens J., Meeusen R. (2015). Effects of caffeine and maltodextrin mouth rinsing on P300, brain imaging and cognitive performance. J. Appl. Physiol..

[28] Cohen J. (1992). A power primer. Psychol. Bull..

[29] Carr A., Dawson B., Schneiker K., Goodman C., Lay B. (2008). Effect of caffeine supplementation on repeated sprint running performance. J. Appl. Physiol..

[30] Evans M., Tierney P., Gray N., Hawe G., Macken M., Egan B. (2017). Acute ingestion of caffeinated chewing gum improves repeated sprint performance of team sports athletes with low habitual caffeine consumption. Int. J. Sport Nutr. Exerc. Metab..

[31] Glaister M., Howatson G., Abraham C.S., Lockey R.A., Goodwin J.E., Foley P., McInnes G. (2008). Caffeine supplementation and multiple sprint running performance. Med. Sci. Sports Exerc..

[32] Kopec B.J., Dawson B.T., Buck C., Wallman K.E. (2016). Effects of sodium phosphate and caffeine ingestion on repeated-sprint ability in male athletes. J. Sci. Sports.

[33] Paton C.D., Hopkins W.G., Vollebregt L. (2001). Little effect of caffeine ingestion on repeated sprints in team-sport athletes. Med. Sci. Sports. Exerc..

[34] Paton C.D., Lowe T., Irvine A. (2010). Caffeinated chewing gum increases repeated sprint performance and augments increases in testosterone in competitive cyclists. Eur. J. Appl. Physiol..

[35] Ranchordas M.K., King G., Russell M., Lynn A., Russell M. (2018). Effects of caffeinated gum on a battery of soccer-specific tests in trained university-standard male soccer players. Int. J. Sport Nutr. Exerc. Metab..

[36] Lee C.L., Cheng C.F., Lin L.C., Huang H.W. (2012). Caffeine’s effect on intermittent sprint cycling performance with different rest intervals. Eur. J. Appl. Physiol..

[37] Lee C.L., Cheng C.F., Astorino T.A., Lee C.J., Huang H.W., Chang W.D. (2014). Effects of carbohydrate combined with caffeine on repeated sprint cycling and agility performance in female athletes. J. Int. Soc. Sports. Nutr..

[38] Kalmar J.M., Cafarelli E. (2004). Caffeine: A valuable tool to study central fatigue in humans?. Exerc. Sport. Sci. Rev..

[39] Davis J.K., Green J.M. (2008). Caffeine and anaerobic performance: Ergogenic value and mechanisms of action. Sports. Med..

[40] Zhang B., Liu Y., Wang X.C., Deng Y.Q., Zheng X. (2020). Cognition and brain activation in response to various doses of caffeine: A near-infrared spectroscopy study. Front. Psychol..

[41] Duncan M.J., Stanley M., Parkhouse N., Cook K., Smith M. (2013). Acute caffeine ingestion enhances strength performance and reduces perceived exertion and muscle pain perception during resistance exercise. Eur. J. Appl. Physiol..

[42] Astorino T.A., Terzi M.N., Roberson D.W., Burnett T.R. (2010). Effect of two doses of caffeine on muscular function during isokinetic exercise. Med. Sci. Sports Exerc..

[43] Endo K., Matsukawa K., Liang N., Nakatsuka C., Tsuchimochi H., Okamura H., Hamaoka T. (2013). Dynamic exercise improves cognitive function in association with increased prefrontal oxygenation. J. Physiol. Sci..

[44] Hogervorst E., Riedel W., Jeukendrup A., Jolles J. (1996). Cognitive performance after strenuous physical exercise. Percept. Mot. Ski..

[45] Kujach S., Byun K., Hyodo K., Suwabe K., Soya H. (2018). A transferable high-intensity intermittent exercise improves executive performance in association with dorsolateral prefrontal activation in young adults. Neuroimage.

[46] Ichinose Y., Morishita S., Suzuki R., Endo G., Tsubaki A. (2020). Comparison of the effects of continuous and intermittent exercise on cerebral oxygenation and cognitive function. Adv. Exp. Med. Biol..

[47] Hogervorst E., Bandelow S., Schmitt J., Jentjens R., Oliveira M., Allgrove J., Carter T., Gleeson M. (2008). Caffeine improves physical and cognitive performance during exhaustive exercise. Med. Sci. Sports Exerc..

[48] Nehlig A. (2010). Is caffeine a cognitive enhancer?. J. Alzheimers Dis..

[49] Souissi Y., Souissi M., Chtourou H. (2019). Effects of caffeine ingestion on the diurnal variation of cognitive and repeated high-intensity performances. Biochem. Behav..

